# The hippocampus and exploration: dynamically evolving behavior and neural representations

**DOI:** 10.3389/fnhum.2012.00216

**Published:** 2012-07-25

**Authors:** Adam Johnson, Zachary Varberg, James Benhardus, Anthony Maahs, Paul Schrater

**Affiliations:** ^1^Department of Psychology, Bethel University, St. Paul, MinnesotaMN, USA; ^2^Department of Computer Science, University of Minnesota, MinneapolisMN, USA; ^3^Department of Cognitive Science, University of Minnesota, MinneapolisMN, USA; ^4^Department of Psychology, University of Minnesota, MinneapolisMN, USA

**Keywords:** hippocampus, memory, place cell, recollection, information foraging, memory consolidation, statistical learning, vicarious trial and error

## Abstract

We develop a normative statistical approach to exploratory behavior called information foraging. Information foraging highlights the specific processes that contribute to active, rather than passive, exploration and learning. We hypothesize that the hippocampus plays a critical role in active exploration through directed information foraging by supporting a set of processes that allow an individual to determine where to sample. By examining these processes, we show how information directed information foraging provides a formal theoretical explanation for the common hippocampal substrates of constructive memory, vicarious trial and error behavior, schema-based facilitation of memory performance, and memory consolidation.

## 1. Introduction

Humans and non-human animals are naturally curious and spontaneously explore (Tolman, 1954; Berlyne et al., 1966; Loewenstein, 1994). A sophisticated set of inference and memory processes inform exploratory behaviors and allow an animal to identify when an observation is “novel” or “surprising” and, as a consequence, warrants exploration (Baillargeon et al., 1985; Ennaceur and Delacour, 1988; Eacott and Norman, 2004; Santos, 2004; Spelke and Kinzler, 2007). More formally, exploratory behavior can be understood as a statistical sampling procedure through which memory representations and inference processes are altered such that past observations are represented more efficiently and future observations become more predictable. Conceptualizing exploration as a statistical sampling procedure leads to the intuitive result that as past observations can be used to better predict future observations, relatively little new information is derived from exploration and exploratory behavior decays.

We identify and discuss two fundamental forms of exploratory activity. The first set of exploratory activities we discuss are experimentally observable behavioral dynamics. In these cases, exploration refers to a behavioral sampling procedure that an animal uses to investigate its environment. The second set of exploratory activities we discuss are representational dynamics that allow an animal to explore previous experience or the inferences available from previous experience. In these cases, exploration refers to a memory-based sampling procedure that an animal uses to investigate a single representation or switch between different representations.

Recent experimental findings suggest that each of these exploratory activities is dependent on the hippocampus and other areas in the medial temporal lobe. The hippocampus appears to support a set of memory processes that allows animals to intelligently and efficiently sample their environments and memory. We call this *directed information foraging*. Directed information foraging has two fundamental components—a process for predicting observations and a process for computing how much new information would be derived from a given observation. The first process is functionally similar to mental imagery (Hassabis et al., 2007b; Schacter and Addis, 2007) while the second process process is functionally similar to the computations that contribute to memory consolidation (Squire and Alvarez, 1995; Nadel and Moscovitch, 1997; Tse et al., 2007). We hypothesize these processes represent fundamental functions of the hippocampus. In the following sections, we review recent findings from the rodent and place cell literatures that show animals engage in directed information foraging and maintain dynamic neural representations in the hippocampus that support exploration through a generative memory process similar to mental imagery.

## 2. Rodent exploratory behavior

**Figure 1:**
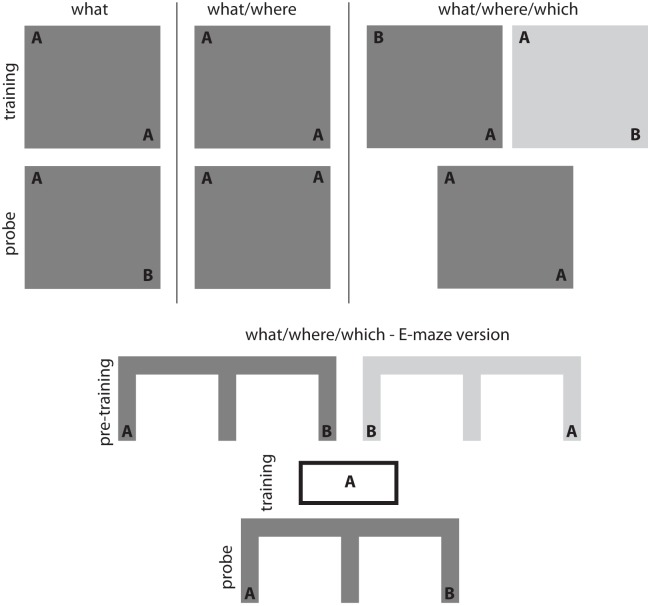
**Exploration-based recognition tasks.** Simple object recognition can be assessed using *what* tasks in which an animal is familiarized with an object during an initial training session. Recognition memory is then assessed by measuring the difference in exploratory behavior associated with a novel object (compared to a familiar control object) during the probe session. Associations between objects and spatial locations can be assessed using *what/where* tasks in which an animal is familiarized with a number of identical objects distributed throughout an arena during an initial training session. Recognition memory for the object-place associations is then assessed by measuring the difference in exploratory behavior associated with the object in a novel position (compared to an object in a familiar control position) during the probe session. More complex associations can be assessed using *what/where/which* tasks in which an animal is familiarized with two different contexts in which objects are distributed throughout the environment. These familiarization periods represent the training period. Recognition memory for these *what/where/which* associations is then assessed by measuring the difference in exploratory behavior associated with an out-of-place object in a particular context. The E-shaped maze version of the *what/where/which* task uses a series of pre-training sessions for acquisition of the *what/where/when* association and then a habituation session with one of the objects (object A in this example). Rats preferentially select the maze-arm with the non-habituated object (object B in the right arm in this example).

Exploratory behaviors have been widely used to study the inferences supported by spatial (Morris et al., 1982; Eacott and Norman, 2004; Day et al., 2003) and non-spatial memory (Ennaceur and Delacour, 1988; Fortin et al., 2002) in rodents. Exploration is informed by specific stimulus information (*what*), spatial location (*where*), contextual information (*which*), observational recency and time of day (*when*) (Ennaceur and Delacour, 1988; Dix and Aggleton, 1999; Eacott and Norman, 2004; Zhou and Crystal, 2009). Within this literature, experimental paradigms that specifically focus on spontaneously initiated exploratory behavior provide a particularly intriguing approach to understanding exploration and its neural substrates. In the simplest version of the spontaneous exploration task (see Figure 1—*what* task), an animal is familiarized with two versions of a single object during a training session. After a delay, the animal is returned to an arena for a probe trial in which one of the two original objects is replaced with a novel object. Recognition memory can be measured by comparing the time spent exploring the novel object relative to the time spent exploring a previously presented object (Ennaceur and Delacour, 1988). Variations on this basic paradigm show that rodents can recognize an object/location pairs (see Figure 1—*what/where* task; Dix and Aggleton, 1999) and the position of an object within a particular context (see Figure 1—*what/where/which* task; Mumby et al., 2002; Eacott and Norman, 2004). Lesion and inactivation studies suggest that spontaneous exploratory behavior in these tasks depends on the medial temporal lobe. Spontaneous exploration on the *what* task requires perirhinal cortex (Bussey et al., 1999; Warburton and Aggleton, 1999; Winters et al., 2004; Winters and Bussey, 2005 but see Ainge et al., 2006), while spontaneous exploration on more complex versions of the task requires the hippocampus (Mumby et al., 2002; Eacott and Norman, 2004).

More recent work using the spontaneous object exploration paradigm has begun to examine the specific memory processes that support exploration. The spontaneous recognition tasks described above cannot distinguish between the contributions of familiarity or recollection to exploratory behavior. Eacott et al. (2005) developed a modified version of the *what/where/which* task to differentiate between the contributions of these memory processes. In the E-maze version of the *what/where/which* task, rats are presented with a spatial choice between a recently encountered object and a less recently encountered object; notably, neither object is observable from choice point (see Figure 1—E-version of the *what/where/which* task). Much like performance on the standard object recognition tasks, rats displayed a novelty preference and preferentially explored the less recently encountered object (Eacott et al., 2005). Preference for the less recently encountered object is impair with fornix lesions but did not impair performance when the object were made visible at the choice point (Easton et al., 2009). Eacott and Easton argue that unlike the standard *what/where/which* task, preferential exploratory behavior on the E-version of the *what/where/which* task cannot be the product of familiarity processes because the objects were not visible from the choice point. Instead, they argue that preferential exploratory behavior on the E-version of the *what/where/which* task must be the product of recollection processes.

Eacott and Easton's work highlights a fundamental difference between familiarity and recollection as sampling processes. Within the spontaneous exploration task, familiarity is a passive sampling process in which an animal simply decides *how long to explore* an object. Determining *how long to explore* requires a comparison of the actual observation and the expected observation. In contrast, recollection is an active sampling process in which an animal must decide *where to explore*. Selecting *where to explore* requires constructing expected observations that would arise from a specific sampling behavior, determining how informative these observations might be, and finally selecting a specific sampling behavior. The differences between familiarity-based and recollection-based sampling behavior can be further developed by a formal treatment of sampling behavior as information foraging.

## 3. Exploration as information foraging: information gain and expected sampling information

In statistical terms, exploratory behavior can be understood as a sampling procedure in which an agent acquires new information. Efficient exploration is equivalent to maximizing the information gained for each sample (Burns and Brock, 2005). Sampling procedures that obtain redundant information are inefficient. Efficient exploration should therefore obtain samples from information rich areas at high densities and information sparse areas at much lower densities. However, in order to know where to sample, the animal must be able to predict where these information rich regions lie. Theoretically, the expected sampling information across a sample space can be computed using the Kullback–Leibler (KL) divergence of a Bayesian prediction. The expected sampling information can then be used to identify the most informative sampling region within a sampling space. In simple terms, maximizing information gain means identifying where the most informative samples can be found.

**Figure 2:**
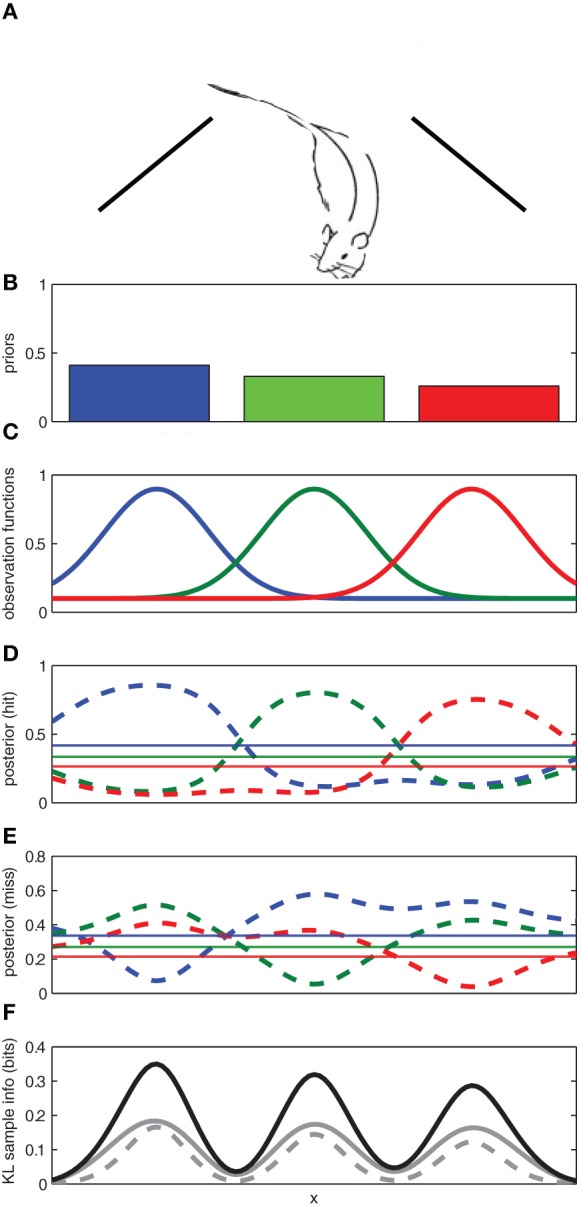
**Using expected sampling information to find an object in a one-dimensional space. (A)** The rat searches along a one-dimensional space (e.g., a wall) for an object. **(B)** The prior probabilities for the left, middle, and right locations. **(C)** The known observation functions for each of the three competing hypotheses for the location of the object. **(D)** The posterior distribution for each hypothesis given an observation of the object (*hit*) at a given location. **(E)** The posterior distribution for each hypothesis given a failure to observe the object (*miss*) at a given location. **(F)** The expected sample information for a hit (*solid gray*), a miss (*broken gray*), and the sum (*black*). The regions with the highest expected sample information across *x* are expected to yield the most informative samples. Here the prior probabilities for each hypothesis were *p*(*h*_1_) = 0.3, *p*(*h*_2_) = 0.2, and *p*(*h*_3_) = 0.5.

To illustrate, let us consider a toy example in which a rat searches for a reward^1^ source along a wall (see Figure 2A). Let us suppose that through previous experience, the rat initially has three competing hypotheses regarding the location of the reward: *h*_1_: the reward source is on the left; *h*_2_: the reward is in the center; and *h*_3_: the reward is on the right. The rat has prior probabilities for each of these hypotheses *p*(*h*_*i*_|*I*) where *h*_*i*_ represents the location of the reward reward source (left/center/right) and *I* denotes previous experience. In order to determine where it should search for the reward, the rat must maintain an observation function for each hypothesized reward location *p*(*y*|*h,I*) that describes probability of observing reward *y* given the assumption that particular hypothesis *h* is true and previous experience *I* (see Figure 2).

Given the observation functions and the priors for each hypothesis, the rat can use Bayes' rule to compute how a given observation (reward present/absent) at any location, *x*, along the wall would affect the probability of each potential reward source (left/center/right)^2^.

(1) $$p(h|y,I)=\frac{p(y|h,I)\;p(h|I)}{\sum_{h^{\prime }}p(y|h^{\prime },I)\;p(h^{\prime }|I)}$$

The posterior distributions for each possible observation outcome are shown in Figure 2. If the observation comes from a particularly informative part of the sampling space, it will cause the hypothetical posterior distribution to diverge from the prior distribution. The divergence between the prior and hypothetical posterior distributions can be used to compute the information gain for each potential observation *y* across the sampling space.

(2) $$Infogain(y)=\sum_{h}p(h|y,I)\log\frac{p(h|y,I)}{p(h|I)}$$

The expected information gain for each potential observation can be computed across the sampling space by weighting the information gain from each possible observation by the probability of the observation. If the expected information gain is computed for each potential sampling location *x* across a sampling space, the expected sampling information is a function of the sample location.

(3) $$KL_{\text{sample info}}(x)=E_{y}\left[\sum_{h}p(h|x,I)\log\left(\frac{p(h|x,y,I)}{p(h|x,I)}\right)\right]$$

As a result, some sampling locations are expected to provide much richer information than others (see Figure 2F). In our toy example, we expect that more information can be gained from sampling at the center of one of reward source locations (left/center/right) than between them.

## 4. Information foraging and memory function

The computations outlined above provide a set of formal distinctions between the processes that contribute to exploration behavior. The sample information (Equation 2) computes the information gain for a single observation. The sample information allows an animal to determine the extent to which a sampling location will continue to yield informative observations. In contrast, the expected sampling information computation (Equation 3) predicts where highly informative observations are expected. Computing the expected sample information allows an animal to direct its exploratory behavior toward information rich areas and sample efficiently. These computations support very different aspects of exploration behavior—sample information supports *undirected* information foraging and expected sampling information supports *directed* information foraging. The following discussion describes the specific behavioral implications of sample information computations and expected sample information computations with an emphasis on the memory and decision processes associated with each computation.

### 4.1. Information gain, undirected foraging, and familiarity

Sample information computes the degree of consistency between an observation and expectations. In our toy example, a “reward absent” observation is more probable than a “reward present” observation (see Figure 2). As a result, a “reward absent” observation provides less informative than a “reward present” observation because it matches expectations (see the dashed gray line panel F). Sample information (information gain) is an information theoretic variant of a prediction error signal that can be used as a learning signal (by gating encoding Hasselmo, 1993). If the sample information (information gain) for a given observation is large, the observation is inconsistent with expectations and learning processes should be initiated; if the information gain for a given observation is small, the observation is generally familiar and minimal learning should occur.

An information gain-based learning signal functionally acts as a familiarity index Yonelinas (2001). Used as a familiarity index, it can be conditioned on variety of dimensions and provides a simple and flexible method for computing familiarity across a variety of representational substrates. For instance an observation can be judged as familiar or not familiar for object identity (*what*), location (*where*), and object identity and location in a given context (*what/where/when*), even time of day. Familiar observations suggest that little information can be gained from further sampling while unfamiliar observations suggest that more information can be gained from further sampling. Sampling unfamiliar observations repeatedly provides the basis for reshape expectations such that sample information decreases. Experimental observations from spontaneous object exploration tasks suggest this familiarization process occurs across the first two or three minutes of standard object recognition tasks (Dix and Aggleton, 1999; Mumby et al., 2002).

Although an information foraging treatment of recognition memory provides a standard set of predictions, it provides several important computational insights. First, sample information computations requires only an observation and expectations associated with the current observation. As a result, sample information operates like a filter for current observations and yields a scalar quantity indicating the information gain for the current sample observation. The properties of the filter are dictated by the statistics of previous experience. Experience-based filtering of current observations is consistent with much research sensory processing including mismatch, novelty, and recency responses observed in temporal cortex and the hippocampus (O'Keefe and Nadel, 1978; Li et al., 1993; Rolls et al., 1993; Zhu et al., 1995; Xiang and Brown, 1998; Brown and Aggleton, 2001; Kumaran and Maguire, 2006) and ROC analysis of familiarity within recognition behaviors (Yonelinas, 2001; Fortin et al., 2004).

Second, the behavioral decision process associated with sample information-based foraging behavior is essentially a go/no-go choice. The animal simply determines *whether to continue* sampling the same stimulus/location/etc. The complexity of the representational substrate is irrelevant to the decision (e.g., *what* versus *what/where* versus *what/where/which*). Exploratory behavior based on this decision process and driven by sampling information is *undirected*: it utilizes only directly observable information and requires no planning. Undirected foraging is driven by a familiarization process in which an animal needs only to pause and attend to high information samples until the sample source is sufficiently familiar to move on to the next sample source.

Given that undirected foraging behavior is supported by sample information computations, a variety of signals should be present within the brain areas that support undirected exploratory behavior. Simple binary decisions such as go/no-go choices can be modeled using integration-to-threshold models (or diffusion-to-bound models; Gold and Shadlen, 2002; Mazurek et al., 2003). Within these models, evidence in support of a particular action accumulates across time until a threshold for action is reached and a specific action is initiated. In undirected information foraging, the two actions are *go—sampling from another location* or *no-go—continue sampling from the same location*. Neural activity associated with integration-to-threshold dynamics has been observed in the lateral intraparietal area and can be used to predict choice behavior and response times (Gold and Shadlen, 2000, 2003; Shadlen and Newsome, 2001; Yang and Shadlen, 2007). Given that simple undirected object recognition is dependent on the perirhinal cortex (Bussey et al., 1999; Winters et al., 2004), we predict that that neurons within perirhinal cortex will display similar integration-to-threshold dynamics that predict go/no-go behavior in simple object recognition tasks. More specifically, the decision process embedded within undirected foraging predicts that a subpopulation of perirhinal neurons will display activity that accumulates (or dissipates) to a standard threshold; once neural activity reaches threshold, the animal will discontinue sampling the current stimulus and begin exploring other aspects of its environment.

### 4.2. Expected sampling information, directed foraging, and recollection

Directed foraging requires an animal to compute the expected sampling information across the sampling space. This computation allows an animal to construct an efficient sampling strategy in order to sample from the most informative regions of a sampling space and avoid less informative regions. As a result, the behavioral decision processes associated with directed information foraging is a *where to sample* choice.

Standard spontaneous exploration tasks conflate directed information foraging with undirected information foraging. An animal might spend more time exploring a novel object because it just stumbled across the object while randomly wandering through the environment—undirected information foraging. Or it might spend more time exploring a novel object because the animal identified the novel object as the most information rich part of the environment and chose to sample it over all other available options—directed information foraging. In order to disambiguate the contributions of undirected and directed information foraging, an experiment must meet two criteria. First, it must force the animal to choose between sampling options with differential expected sampling information. Second, it must prevent apparent exploration that is the product of randomly stumbling into the highly informative region of the sampling space^3^.

The E-maze version of the *what/where/which* task meets each of the experimental criteria for assessing directed information foraging. The E-maze version of the *what/where/which* task has three phases (see Figure 1 above). In the first phase, a rat receives a series of training sessions in which it learns the location of two objects within the E-maze. The training sessions are a critical component of the task because they allow the animal to form expectations about the observations available within each maze arm. In the second phase, the rat is given a habituation session with one of the two objects. This devalues the informativeness of the object and makes it “less novel.” In the third phase, the rat is presented with a choice between maze arms leading toward the “less novel” and the “more novel” object. Rats typically display a novelty preference and choose the path toward the “more novel”, non-habituated object. The effect persists even when the objects are not visible from the choice point. Because the objects are not visible from the choice point, the animals cannot use undirected information foraging and must make their choice according to the expected sampling information associated with each option.

**Figure 3:**
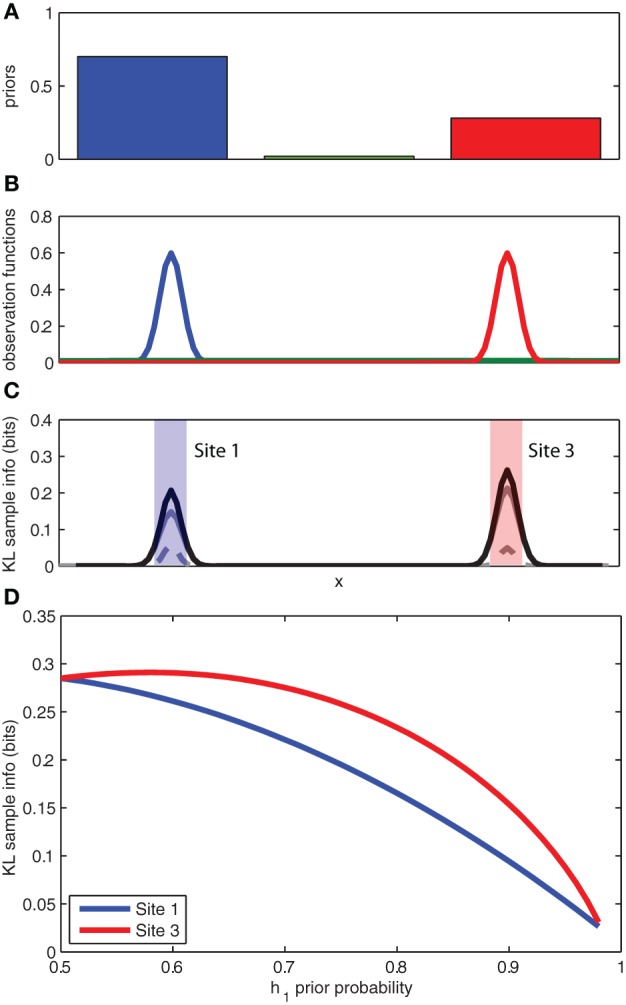
**Novelty preferences as information foraging. (A)** The prior probabilities for the left, middle, and right locations. **(B)** The known observation functions for each of the three competing hypotheses for the location of the object. **(C)** The expected information gain as calculated by the KL divergence for a hit (*solid gray*), a miss (*broken gray*), and the sum (*black*). **(D)** The expected information gain (bits) for sampling at the left location (site 1) and the right location (site 3) as a function of the prior probability associated with the left location (site 1). See panel **C** for left and right locations. Note that expected information gain is higher at the site opposite to the higher prior probability.

The experimental observation that animals prefer to attend to or search out unexpected stimuli, even when these stimuli may not be directly observable, can be explained by directed information foraging. In Figure 3 we return to our toy example of a rat searching for a reward source along a wall. The observation functions have been modified to reflect two highly probable reward source locations and a third highly improbable source location (see Figure 3B). The expected sampling information for each location is shown in Figure 3C. Because the expected sampling information is dependent on the priors, we can plot the expected sampling information at each feeder location (*left/right*) as a function of the prior probability for a given feeder location (see Figure 3D). Predictably, the expected sampling information decreases as the animal becomes more certain of the active feeder location (e.g., the prior probability for a given active feeder *p*(*h*_1_) → 1). However, an interesting aspect of the expected sampling information computation is that more information is expected from sampling at the feeder location that has the lower prior probability. This suggests that directed information foraging naturally produces a novelty preference.

Eacott and Easton contend that because an animal must make its choice on the E-maze version of the *what/where/which* task according to an *expected* observation, the animal must *generate* this observation from memory (Eacott et al., 2005). Although they argue this generative process entails recollection (Eacott et al., 2005; Easton and Eacott, 2008), the animal could simply access a set of stimuli associations rather than engaging in a true recollective process. The expected sampling information computation similarly requires generating the likelihood of potential observations across the sampling space—a computation that again can either be performed by generating the outcome on the spot or retrieving the probability of outcomes from a cache. Two experimental observations suggest that rat indeed generate observations according to a true recollective process. First, lesions of the hippocampus compromise novelty preference on the E-maze version of the *what/where/which* task (Eacott et al., 2005; Easton and Eacott, 2010). Second, ROC analysis of recognition memory shows that rats with hippocampal lesions display behavior that is consistent with a loss of recollective memory processes (Fortin et al., 2004; Eichenbaum et al., 2007). The deficits caused by hippocampal lesions in rats are also consistent with human patients with compromised episodic memory and suggest that recollective memory retrieval is governed by a binary successful generation of failed generation of an observation (Yonelinas, 2001).

#### 4.2.1. The evolution of directed information foraging

The development of directed information foraging is the product of two distinct learning processes. The first learning process is associated with the development of observation functions. Observation functions indicate the conditional probability of making an observation (reward present/absent) at any sampling location *x* given a particular source location (the active feeder positioned on the *left/center/right*). The second learning process is a discriminative learning process associated with the differential development of priors associated with each source location (*left/center/right*).

**Figure 4:**
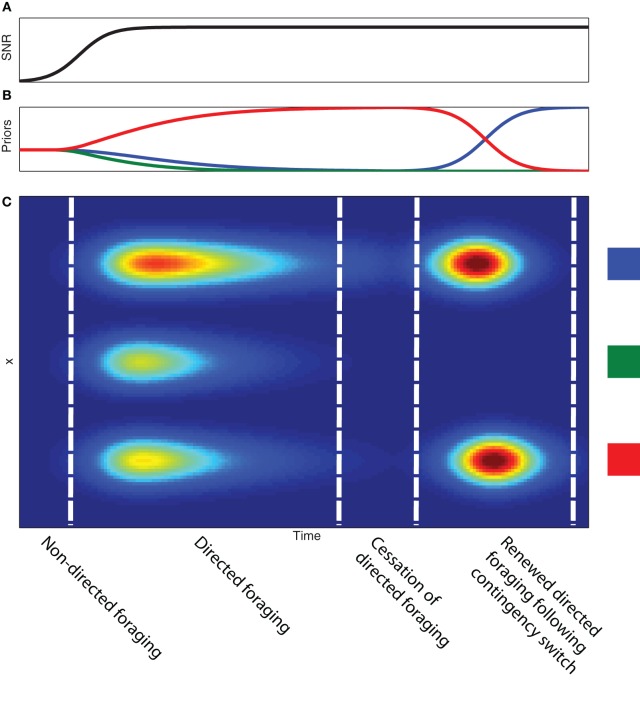
**Development of information foraging with experience. (A)** Developing signal-to-noise ratio of the observation function as a function of time. **(B)** Developing prior probabilities as a function of time. **(C)** Expected information gain as a function of time (x-axis) and sample position (y-axis). Note the development of higher expected information coincides with the development of the signal-to-noise ratio of the observation functions. The uniform expected information gain suggests an initial period of non-directed or random foraging behavior. The non-uniform expected information gain allows the animal to transition into a period of directed foraging across the three feeder sites. Although the development of differential expected information gain is the result of the prior probabilities associated with each site, increasing the prior probability of a single site to near certainty produces decreased expected information gain across the entire sampling space. This produces a cessation of information foraging. A contingency switch produces a change in the prior probabilities (in this case between the blue and the red feeders) and leads to another bout of information foraging.

In Figure 4, we show how directed information foraging develops as a function of evolving observation functions and differential development of priors. We model the development of observation functions as gaussian distributions contaminated by a uniform noise function (see Figure 2). As learning occurs, the signal-to-noise ratio—the ratio of the amplitude of the gaussian function to the amplitude of the uniform noise function—for each of the observation functions increases (Figure 4A). The development of the observation functions provides the basis for a transition from random sampling behavior to directed sampling behavior that is focused on the three source locations. Figure 4C shows the differential development of expected sampling information across the sampling space even when the priors associated with each of the three source locations are uniformly distributed. Differential development of the priors associated with each of the three source locations leads to differential expected sampling information at each of the source locations. If evidence accumulates in support of a single “winning” source location, expected sampling information decreases across all sampling locations and directed information foraging ceases^4^. The evolution of observation function and priors leads to a specific sequence of foraging behavior during learning: (1) initial random foraging, (2) developing directed foraging, (3) cessation of directed foraging and a transition to exploiting reward-related information (if reward is present).

An example of this kind of increasingly specific foraging behavior has been studied by Morris and colleagues using the paired-associated task (Day et al., 2003; Tse et al., 2007; Bethus et al., 2010). In the paired-associate task, rats learn that a specific flavor indicates the location of reward among a matrix of food locations. The flavor/location association is called a paired associate and different flavors indicate reward at different locations. Rats learn the initial set of paired-associates slowly over a series of daily training sessions spread across several weeks. However, acquisition of new paired-associates following initial learning requires as little as a single paired-associate presentation (Tse et al., 2007). Morris and colleagues explain single trial learning in terms of developing task schemas that facilitate learning and subsequent behavioral performance by focusing exploratory behavior.

This interpretation provided by Tse et al. (2007) suggests that performance on the paired-associate task is governed by two distinct learning processes: schema learning in which the animal learns that flavors predict specific reward locations and discriminative associative learning in which the animal learns which flavor is associated with which reward location. These learning processes directly correspond to the two learning processes embedded within directed information foraging. In our toy example, schemas correspond to observation functions and discriminative associative learning corresponds to the beliefs mediated by the prior and the posterior distributions. Just as the development schemas facilitate learning by focus search behavior and learning more from a single observation, the development of observation functions directing search behavior toward highly informative observations and increase the impact of these observations.

#### 4.2.2. Vicarious trial and error

The temporal development of directed information foraging is reminiscent of the development of vicarious trial and error (VTE) behavior on discrimination tasks described by Muenzinger (1938) and Tolman (1939). VTE behavior occurs when an animal pauses at a choice point and orients toward different possible spatial options before making a decision. Tolman (1939, 1948) argued that an animal vicariously samples the outcome of each option during VTE behavior. VTE is then a form if directed information foraging in which an animal samples memory rather than a physical space and VTE behavior is the observable residual of sampling from a memory.

Tolman (1939, 1948) described the development of VTE behavior in three phases. The first phase of behavior on tasks in which VTE behavior occurs is simple trial and error behavior characterized by random sampling of different choice options. VTE behavior is absent during this initial phase. The second phase of behavior is punctuated by high levels of VTE behavior at choice points and increasing performance on memory or discrimination tasks. During the third phase of behavior, memory or discrimination performance increases to ceiling and VTE behavior ceases.

VTE behavior occurs on a variety of choice tasks and can be induced by altering task contingencies (Tolman, 1939; Blumenthal et al., 2011). On tasks in which VTE behavior is induced through contingency changes, VTE behavior occurs at specific, highly informative task locations (Blumenthal et al., 2011) including the the choice point on the E-version of the *what/where/when* task (Alexander Easton, pers. comm.).

Directed information foraging can be used to simulate and predict VTE behavior that is induced by a change in task contingencies. In our toy example, we model a change in task contingency by modifying the prior beliefs regarding which feeder is active. If the priors associated with a food source at on the left and right switch (as indicated by the red and blue curves on the right side of Figure 4A), reflecting a contingency reversal, the expected sampling information increases and information foraging begins again. Learning higher order task contingencies allows foraging to be more precisely directed to highly informative sampling areas. We believe that VTE behavior reflects the use of learned higher order task contingencies to simulate or imagine the outcomes of different behavioral sampling. If true, VTE dependents on at least a rudimentary form of mental imagery.

And like recollection-based performance on the E-version of the *what/where/when* task, VTE behavior is dependent on the hippocampus (Hu and Amsel, 1995) and drives increased metabolic activity in the hippocampus (Hu et al., 2006). Moreover, the development of task schemas that support increasingly specific directed foraging are also hippocampus dependent (Tse et al., 2007). These converging experimental results highlight the various roles of the hippocampus in directed information foraging.

From its initial treatment by Tolman (1939), VTE has encountered a variety of conceptual and experimental challenges. Guthrie (1952), for instance, critiqued Tolman's description of the mechanisms that support VTE behavior, suggesting that his theory left rats “buried in thought” when confronted with a choice. This conceptual criticism continues to plague theoretical treatments of VTE. Experimentally, VTE presents a variety of challenges associated with defining orienting behavior at choice points and its frequently transient presence in most tasks. For these reasons, VTE has remained sparsely studied over the past seventy years.

Information foraging provides a conceptual framework for both developing a theory of VTE and future experimental investigations of VTE behavior. The dynamics of information foraging suggest a novel approach to analysis of the transient and often subtle sampling behavior observed in VTE behavior. The computational requirements of information foraging—recollection-like processes associated with generation of potential observations that are shaped by task schema—and its connection to reinforcement learning algorithms such as POMDP address Guthrie's long-standing conceptual critique. Converging evidence from hippocampal lesion studies on VTE behavior (Hu and Amsel, 1995), recollection (Easton et al., 2009), and schema development (Tse et al., 2007) provide an experimental path toward understanding the neural substrates of VTE behavior as well.

### 4.3. Conclusions: behavioral exploration

The previous discussion has shown how information foraging can be used to differentiate two major classes of exploratory behavior. Undirected information foraging is characterized by a *go/no-go* decision process based on the sample information (e.g., familiarity) and depends on extra-hippocampal areas, principally the perirhinal cortex. In contrast, directed information foraging is characterized by a *where to go* decision process based on expected sampling information and depends on the hippocampus. While the *where to go* decision process is most frequently a spatial question (O'Keefe and Nadel, 1978), the key contribution of the hippocampus is generating potential observations using a constructive recollective memory process. This generative process utilizes hippocampus-dependent schemas in order to more precisely direct foraging behavior to highly informative samples (Tse et al., 2007).

Information foraging provides a formal approach that shows how hippocampal-dependent schemas and recollective processes interact. It provides a clear, quantitative approach that allows precise analysis of exploratory behavior, both in terms of its distribution of spatial sampling and in terms of its temporal evolution. Finally, it provides, for the first time, a comprehensive theory of vicarious trial and error behavior.

## 5. Exploring memory

We now discuss the representational dynamics that allow an animal to explore memory. Memory-based information foraging allows an animal to obtain samples from its memory rather than its environment. Memory-based exploratory activity, much like behavioral exploratory activity, can be split into undirected and directed information foraging. Animals can stumble across informative memories—undirected foraging; or they can actively search for them—directed foraging. Directed information foraging applied to memory-based information foraging provides a formal approach to understanding how an animal locates particularly useful and informative memories within its memory (even when these aren't “strong” memories). The following discussion focuses on memory-based directed information foraging.

### 5.1. Hippocampal representational dynamics

We identify two major dynamics observed in hippocampal neural activity that support directed information foraging. The first hippocampal dynamic we discuss is hippocampal sweeps (Johnson and Redish, 2007; Gupta, 2011). Hippocampal sweep dynamics allow an animal to sample different spatial locations from memory. This VTE-like dynamic supports the “vicarious” sampling process embedded within vicarious trial and error behavior. The second hippocampal dynamic we discuss is hippocampal map-switching (Jackson and Redish, 2007; Fenton et al., 2010; Kelemen and Fenton, 2010). The map switching dynamic allow an animal to re-represent its current task with respect to different reference points. Each of these representational dynamics provide the animal with an opportunity to obtain maximally informative information from memory.

#### 5.1.1. Hippocampal sweeps

Directed information foraging predicts that animals will sample from memory as VTE behavior occurs. We expect that VTE behavior and the representational dynamics that support VTE occur at points within a task where simple familiarity fails to provide adequate information and further retrieval is required to make an informed choice. This predicts the locations when sweep dynamics should occur within a task. We further expect that the information retrieved during VTE to reflect highly informative aspects of the task that will, in turn, contribute the animal's choice behavior. This predicts what or where sweep dynamics should represent within a particular task.

**Figure 5:**
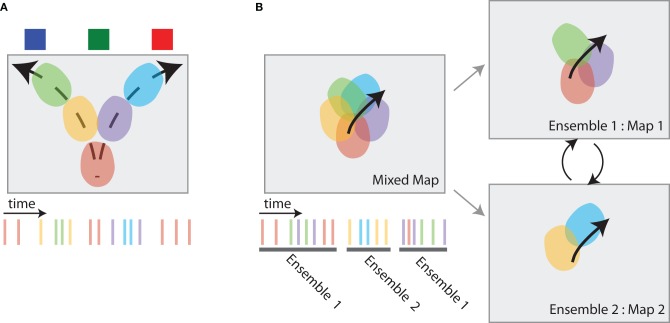
**Hippocampal dynamics associated with directed information foraging. (A)** VTE-like dynamics in the hippocampus are non-local representations that occur at choice points. While the animal pauses at the choice point (the bottom-center of the box), spatial representations in the hippocampus move ahead of the animal, following potential trajectories toward the different reward source locations. The spiking activity associated with these place cell dynamics is shown at right. **(B)** Map-switching is a representational dynamic in which place cell ensembles alternate between two maps. An initial map of place fields (left, top) can be divided into different maps (right) by identifying two or more sets of temporally non-overlapping ensemble spiking activity (left, bottom). These maps often reflect different task components.

Johnson and Redish (2007) trained rats on a sequential spatial decision task in which VTE is observed (Johnson and Redish, 2007; Blumenthal et al., 2011). Much like the findings from previous studies, hippocampal place cells usually display spiking activity as the animal runs through each cell's place field on this task. Such “within field” activity is consistent with the notion that the hippocampus represents the animal's current position as it moves through the maze. However, place cells also displayed “out of field” spiking activity at the high cost choice point on the maze. Johnson and Redish (2007) found that “out of field” spiking at the choice point was coordinated across the ensemble; decoding the animal's position during epochs of high “out of field” spiking at the choice point predicted coherent position estimates that dynamically moved from the animal's current position at the choice point toward feeder locations (see Figure 5A). These hippocampal dynamics are consistent with memory retrieval processes embedded within VTE and the dynamics predicted by directed information foraging: they occur when the animal encounters a high cost choice and insufficient information is available from familiar environmental cues *and* they represent positions ahead of the animal that correspond to future potential trajectories.

#### 5.1.2. Map switching

Directed information foraging predicts that animals will dynamically shift between competing task representations and will utilize a task representation that best predicts environmental observations. In the two-frame avoidance task used by Kelemen and Fenton (2010) rats must maintain representations of their current position two different reference frames in order to avoid a shock. One reference frame is based on stable room cues and predicts the location of an otherwise invisible shock zone. The second reference frame is based on local cues embedded within the navigation arena and predicts the location of another invisible shock zone. Avoidance behavior on this task is dependent on the hippocampus (Cimadevilla et al., 2001; Wesierska et al., 2005; Kelemen and Fenton, 2010). Place cell activity within the two-frame avoid task reflects both the room and arena reference frames: one set of place cells forms a stable map within room frame and a second set of place cells forms a stable map within the arena frame (see Figure 5B *right*). Place cell activity within each maps maintain an estimate of the animal's current location within that map. However, ensemble activity coherently switches between reference frames (see Figure 5B *left*). Kelemen and Fenton (2010) showed that the hippocampal map that better predicts the proximal shock zone is consistently more active. When the shock zone associated with the room reference frame is closer to the animal, place cells that represent the animal's location in the room reference frame are more active; when the shock zone associated with the arena reference frame is closer to the animal, place cells that represent the animal's location in the arena reference frame are more active.

The hippocampal dynamics found on the two-reference frame avoidance task can be understood as directed information foraging from memory. At each moment, the hippocampus can represent the animal's current position in one of two competing maps. Within the two-reference frame avoidance task, the expected sampling information associated with each reference reference varies across time. The expected sampling information associated with each reference frame is a function of the animal's location within each reference frame and the observations the animal would expect as a result of its locations within the reference frame. Given a gradual accumulation of noise in the hippocampal estimate of the animal's location within each reference frame, directed information foraging allows the animal to most efficiently update its location across reference frames. More specifically, directed information foraging suggests that the animal should activate the map that will yield the richest and most task salient observations at a particular location so that discrepancies between the animal's current estimate of its location and its actual location (as indicated by available observations) can be found.

### 5.2. The temporal evolution of representational dynamics

We propose that the temporal evolution of these hippocampal dynamics parallels the development of directed information foraging behavior outlined above (see Figure 4). Initially, these hippocampal representational dynamics are absent because observation functions and possible task schema have not been learned. As the animal learns the observation functions and possible task schema, these hippocampal representational dynamics develop and reach their peak frequency. Finally, if the animal learns the task well enough to predict task-related observations, hippocampal representational dynamics provide no additional information and, consequently, diminish in frequency. However, if the task-related observations remain difficult to predict, hippocampal representational dynamics continue to provide important task-related information and continue to occur at a high frequency.

The observed temporal evolution of VTE-like representational dynamics in the hippocampus is consistent with directed information foraging from memory. Johnson and Redish (2007) showed that hippocampal sweeps increase in frequency during early behavior and diminish as animals are able to predict the outcomes of a simple spatial choice. However, the frequency of hippocampal sweeps did not appear to diminish on a cued version of the task in which the outcomes of a spatial choice were much more difficult to predict (Johnson and Redish, 2007). Similarly, map switching dynamics on the two-frame reference task (Kelemen and Fenton, 2010) and similarly complex tasks (Jackson and Redish, 2007) occur at high frequency but diminish in frequency on simple tasks (Jackson and Redish, 2007; Fenton et al., 2010).

### 5.3. Directed information foraging and memory consolidation

We believe that the temporal evolution of directed information foraging provides deep insights into memory consolidation. The representational dynamics that support directed information foraging from memory are based on generating/retrieving information that is not otherwise available from simple associative (familiarity-based) memory processes. Given that the function of directed information foraging is to provide a set of observations that will most greatly alter simple associative learning processes, directed information foraging diminishes when simple associative learning mechanisms can support behavioral performance. In these cases, directed information foraging is unnecessary and task performance is independent of the neural substrates that support directed information foraging. This is a functional description of memory consolidation.

A variety of memory tasks display a temporally limited dependence on the hippocampus (Zola-Morgan and Squire, 1990; Packard and McGaugh, 1996; Teng and Squire, 1999; Maviel et al., 2004; Morris, 2006; Tse et al., 2007, 2011). In many of these tasks, behavior that is initially dependent on the hippocampus becomes dependent on frontal cortices as task information is consolidated to these non-hippocampal areas (Maviel et al., 2004; Tse et al., 2011). Although a variety of theories attempt to explain consolidation in terms of differential learning rates across different brain areas (medial temporal lobe encoding is fast and obligatory while neocortical encoding is slow and selective; Squire and Alvarez, 1995; Nadel and Moscovitch, 1997), recent findings by Morris and colleagues suggest that previous learning can facilitate consolidation (Tse et al., 2007, 2011; Bethus et al., 2010). Tse et al. (2007) showed that following the initial training period on the paired-associate task, hippocampus dependent single trial learning underwent consolidation within 48 h. Explaining these findings, Tse et al. (2007) suggest that schema learning facilitated consolidation but leave open the specific mechanisms that facilitate consolidation.

Time varying consolidation can be understood in terms of information foraging. Sampling behavior during initial learning is based on random foraging and provides relatively uninformative samples that slowly reshape simple associative learning processes outside the hippocampus. Sampling behavior during later learning is based on directed information foraging and provides highly informative samples that quickly reshape simple associative learning processes outside the hippocampus. As a result, learning that occurs after the animal has learned relevant task schemas can utilize directed information foraging and train non-hippocampal learning processes more quickly, thereby making behavioral performance less dependent on the hippocampus more quickly.

Treatment of memory consolidation within the context of directed information foraging leads to the intriguing prediction that the consistency of place cell activity could be used to predict the hippocampal dependence of a task. In order to more thoroughly develop this prediction, consider how representational dynamics in the hippocampus lead to increased levels of apparent noise within place cell activity (see Figure 5; Johnson et al., 2009)^5^. Given our previous description of the task-dependent temporal evolution of directed information foraging and its associated hippocampal dynamics (see the previous subsection), we predict that on tasks where memory consolidation occurs, place cells will display high levels of apparent noise and instability followed by a reduction in apparent noise and increased place cell stability across task acquisition. In contrast, we predict that on tasks where memory consolidation does not occur, place cells will display high levels of apparent noise and instability across task acquisition. As a result, we predict that hippocampus independent behavioral performance in individual animals with low levels of apparent place cell noise after task acquisition^6^.

### 5.4. Conclusions: memory exploration

The previous discussion has shown how directed information foraging can be extended to generative memory dynamics in the hippocampus. Memory-based directed information foraging suggests that hippocampal sweep dynamics and map switching dynamics are based on an active search for information from hippocampal memory. Directed information foraging suggests when these dynamics should occur within a behavioral task and what information is represented by these transient dynamics. Both when generative memory dynamics occur and what information they represent is governed by developing task representations and schema.

The temporal evolution of hippocampal memory-based directed information foraging mirrors the temporal evolution of directed information foraging behavior. Memory-based directed information foraging comes to an end when simple associative memory processes predict all task relevant observations and, consequently, can support task performance. As a result, the cessation of memory-based directed information foraging signals the transition from recollective memory processes to simpler associative process and memory consolidation. Finally, because memory-based directed information foraging and the hippocampal dynamics that support it are associated with increased apparent noise in place cell activity, directed information foraging predicts that apparent noise can be used to gauge memory consolidation and the developing hippocampal independence of task performance.

## 6. Control processes in directed foraging

Behaviorally observable and covert sampling behaviors are the product of decision processes. Our previous discussion suggests that the information available within the hippocampus is used to control both observable and covert sampling behavior. We hypothesize that medial prefrontal cortex (mPFC) plays a central role in the control of both overt behavioral sampling behavior and covert mnemonic sampling within hippocampal representations.

The mPFC has been widely implicated in the flexible control of behavior (Granon and Poucet, 1995; Balleine and Dickinson, 1998; Corbit and Balleine, 2003; Ostlund and Balleine, 2005; de Wit et al., 2006; Rich and Shapiro, 2007) and memory retrieval (Maviel et al., 2004; Churchwell et al., 2010; Tse et al., 2011). We hypothesize that the mPFC functions as a controller that utilizes hippocampus-based expected sampling information signals to direct behaviorally observable information foraging and retrieval processes that support covert information foraging. The control processes associated with directed information foraging within the mPFC are dependent on the development of hippocampal representations that allow the mPFC to select *where* to sample in order to maximize information gain. During initial learning when hippocampal representations are relatively poorly developed, mPFC utilizes expected information gain signals from the hippocampus to direct behavioral sampling. As task learning progresses and hippocampal representations are better developed, mPFC utilizes expected sampling information signals from the hippocampus to direct retrieval processes that support covert information foraging. Finally, as expected information signals from the hippocampus based on retrieval processes decay, memory consolidation occurs and renders retrieval processes hippocampus independent. As result, our proposal suggests that mPFC lesions will produce both behavioral deficits such as reduced VTE behavior and absence of hippocampal dynamics associated with directed information foraging.

Several recent studies support our proposal. Tse et al. (2011) recently showed that paired associate learning activates both mPFC and hippocampus when previously learned schemas can contribute to learning, but learning only activates the hippocampus when no previously learned schemas can contribute. Moreover, temporary lesions of mPFC compromised retrieval on the paired-associate task. If mPFC lesions compromise hippocampus-based memory retrieval, we expect that hippocampal activity associated with retrieval processes will be reduced and consequently increase observed consistency within place cell activity. Consistent with this prediction, Kyd and Bilkey (2003) showed that the information content of place cells increases following mPFC lesions.

If mPFC neurons support the control processes necessary for directed information foraging, we predict three classes of mPFC neural activity should emerge during directed information foraging. The first class of mPFC neurons codes the expected sampling information available within a task and provides the basis for directed foraging behavior. We predict that these neurons code specific sampling strategies and behavioral sequences. The second class of mPFC neurons code the expected sampling information available in memory and provides the basis for directed foraging from memory. These neurons control hippocampal retrieval dynamics such as hippocampal sweeps (Johnson and Redish, 2007). We predict that these neurons code specific memory sampling strategies and mnemonic sequences that dictate the extent of hippocampal retrieval. The third class of mPFC neurons code a statistically compact task representation. We predict that these neurons, in tandem with the second class of mPFC neurons, support memory consolidation. A statistically compact task representation is a representation for which expected sampling information is minimized and, as a result, the memory is stabilized. For example, we predict that as an animal makes different observations which provide redundant task information, this information will be represented categorically.

These predictions are consistent with a variety of recent findings from mPFC recording studies. mPFC neurons differentially code sampling strategies—even while the animal performs the same behavior—when strategies must be used to solve a strategy-based plus maze task (Rich and Shapiro, 2009). And a subset of prelimbic mPFC neurons increased firing rates following changes in task contingencies on the plus maze and returned to baseline firing rate as performance returned to asymptotic levels with subsequent learning. These observations are consistent with our prediction that neural activity within mPFC codes for the expected sampling information available within the task—a signal that can be used to inform overt directed information foraging behavior.

Although few studies have explicitly examined mPFC-mediated hippocampal retrieval in rodents, a number of studies have identified coordinated theta activity between the hippocampus and mPFC that may support information transfer between these areas (Jones and Wilson, 2005; Siapas et al., 2005; Adhikari et al., 2010). We predict that theta synchrony between the hippocampus and mPFC are associated with behavioral information foraging while transient reductions in theta coherence are associated memory retrieval processes associated and covert information foraging. Adhikari et al. (2010) showed that the coherence theta oscillations in mPFC and ventral hippocampus decreases immediately before the animal enters the high anxiety arm of the elevated plus maze. During these periods, animals display behaviors very similar to VTE (Kaesermann, 1986) and the spiking activity of a subset of mPFC neurons represent the future position of the animal rather than the animal's current sensory cues (Adhikari et al., 2011). These observations are consistent with our prediction that that the presence of coordinated theta activity across the mPFC and hippocampus is associated with behaviorally observable directed information foraging while transient reductions in mPFC-hippocampal theta activity are associated with the retrieval processes that support covert information foraging from memory.

Finally, many studies suggest that mPFC neurons flexibly code salient sensory information, behavior, and goals (Hok et al., 2005; Hyman et al., 2005, 2010; Cowen and McNaughton, 2007; Rich and Shapiro, 2009; Adhikari et al., 2011). Although ascertaining the extent to which mPFC neurons code compact task representation is currently experimentally challenging, Adhikari et al. (2011) have shown that spiking activity in mPFC neurons on the elevated plus maze represent non-redundant task information. Future work is needed to determine how mPFC representations develop and support memory consolidation.

## 7. Connections with human memory research

Our review has focused on recent developments within the rodent literature highlighting the contributions of the hippocampus and medial temporal lobe structures to exploratory activity. We used information foraging as a formal framework to examine the fundamental computations and mechanisms that support exploratory behavior in rodents. However, information foraging can also be applied to recent work on human exploratory behavior (Hartley et al., 2003; Voss et al., 2011b,c), the contribution of metacognition to individual study patterns (Dunlosky and Hertzog, 1998; Metcalfe, 2009), and constructive episodic memory (Buckner and Carroll, 2007; Hassabis et al., 2007b; Schacter et al., 2007).

### 7.1. Human information foraging

A particularly intriguing series of experiment by Voss and colleagues (Voss et al., 2011a,b,c) explicitly investigated human information foraging. Subjects were asked to study a two-dimensional grid of images and commit each image and its location to memory. In the volitional control condition, subjects controlled the location of the viewing window and actively explored the objects in the environment in order to learn object/location pairs. In a passive viewing condition, subjects were presented with the views selected by the previous subject and were not able to actively explore the environment. From an information foraging perspective, the key difference between the two conditions is that subjects in the yoked-control condition were unable to engage in directed information foraging behaviors.

Subjects performed better on subsequent memory tests in the volitional control condition compared to the passive control condition. Furthermore, subjects in the volitional control condition reported higher levels of remembering, a process indicative of recollection, than subjects in the passive viewing condition. Subjects in the volitional control condition also spontaneously revisited previously viewed object/location pairs, an explicit information foraging behavior that resulted in increased subsequent memory performance even after controlling for viewing time. These results suggest that subjects in the volitional control condition engaged in directed information foraging and were able to obtain more informative viewing samples with respect to their specific schemas and memory content.

Subjects with damage to the hippocampus displayed little directed information foraging behavior in the volitional control condition and none of the memory gains associated with volitional control observed in hippocampal controls subjects. Volitional control activated a prefrontal-hippocampal-parietal network while specific information foraging behaviors (spontaneous revisitation) were associated with more specific activation of the left anterior hippocampus and left medial frontal gyrus.

The findings by Voss and colleagues (Voss et al., 2011a,b,c) provide an important link between directed information foraging behaviors in rodents and humans. Spontaneous revisitation behavior is functionally equivalent to the novelty preference observed on the E-maze version of the *what/where/which* task: subjects in the volitional control condition must make a spatial choice between stimuli and prefer novel stimuli with respect to their current memory traces. Such behavior is based on an assessment of expected sampling information computation. Moreover, drawing insights from the rodent literature, we expect that eye-tracking in the volitional control condition would most likely reveal VTE-like glances across different spatial locations that allow a subject to assess memory strength and decide whether revisitation is necessary or beneficial.

### 7.2. Metacognition and information foraging

In order to engage in information foraging, an individual must ascertain whether information can be gained by sampling. Directed information foraging further suggests that an individual must anticipate the relative information gain across a set of sampling behaviors in order to decide where to sample. Such evaluations are closely linked with metacognitive judgments that are used to determine how much an individual should study (Dunlosky and Hertzog, 1998; Metcalfe and Kornell, 2005). In fact, the information theoretic approach to directed information foraging outlined above provides a normative statistical basis for the region-of-proximal-learning framework (Metcalfe and Kornell, 2005; Metcalfe, 2009). This framework predicts that individuals will allocate study-time according the relative rate of information gain and that they will stop studying once the rate of information gain reaches zero. Although humans clearly compute these expected information gain evaluations across much more sophisticated representational content than do animals, the computations that underlie such information theoretic predictions are, in principle, identical^7^.

### 7.3. Constructive episodic memory and information foraging

Constructive episodic memory can be understood as covert information foraging within a memory space rather than an explicitly spatial foraging space as used by Voss et al. (2011b,c). Constructive episodic memory prompts require more information than can be provided by associative processes within semantic memory (Addis et al., 2007; Hassabis et al., 2007b). These prompts increase the expected sampling information associated with a given position in an episodic memory space. Sampling from memory space is analogous to episodic memory retrieval. As a result, episodic memory retrieval and directed information foraging should inform autobiographical search behaviors such as looking through a family album.

The control processes that regulate constructive episodic memory have only recently begun to be studied (Buckner and Carroll, 2007; Hassabis et al., 2007a; Schacter et al., 2007; Summerfield et al., 2010). Guthrie's critique of vicarious trial and error, specifically that it leaves rats “buried in thought” (Guthrie, 1952), can be similarly asked of constructive episodic memory: how does an individual know when to stop searching through memory and respond to the prompt? Information foraging provides a formal approach that follows the basic intuition that retrieval processes embedded within constructive episodic memory cease when no further memory content can inform a response.

## 8. Conclusions

Information foraging represents an ever increasing part of daily life. Our formal treatment of exploration as information foraging highlights the specific processes that contribute to active, rather than passive, exploration and learning. We hypothesize that the hippocampus plays a critical role in active exploration through directed information foraging by supporting a set of processes that allow an individual to determine *where* to sample. The directed information foraging approach to hippocampal function is consonant with previous explanations of hippocampal function as fundamentally spatial (O'Keefe and Nadel, 1978; Redish, 1999); however, our approach connects spatial conceptions of hippocampal function with more general memory-based approaches to hippocampal function (Eichenbaum et al., 1999; Squire et al., 2004). Directed information foraging provides a formal theoretical explanation for the common hippocampal substrates of constructive memory, recollection, schema-based facilitation of memory, and memory consolidation. We leave further elaboration of the directed information foraging framework to future research but note its utility in constructing specific behavioral predictions with respect to search behavior and analyzing transient hippocampal dynamics.

### Conflict of interest statement

The authors declare that the research was conducted in the absence of any commercial or financial relationships that could be construed as a potential conflict of interest.

## Footnotes

^1^The example can be extended to any type of observation and to any sampling space (e.g., a physical set of locations, a set of actions, or a memory space).

^2^Technically, the sample space and the sampling procedure should be included in this calculation. That is, likelihood and prior should be conditioned on the sample space and sampling procedure. We neglect these for simplicity.

^3^This can be accomplished by preventing the animal from obtaining sample prior to making its choice.

^4^Note that if reward is embedded within the task, the animal may continue to exploit that knowledge.

^5^VTE-like representational dynamics increase apparent noise within place cell activity because place cells transiently represent non-local positions in the environment rather than the animal's current position in the environment. Map switching dynamics increase apparent noise within place cell activity because place cells that are active at a one spatial location on one map are not active at the same spatial location on another map.

^6^Apparent noise in place cells can be identified using measures like overdispersion (Fenton et al., 1998, 2010; Jackson and Redish, 2007). Jackson and Redish (2007) and Fenton et al. (2010) have measured overdispersion on hippocampus dependent open field foraging tasks to be approximately 3.2–3.8. This value decreases to approximately 2.0 on hippocampus independent tasks such as the linear track.

^7^Studies on the contribution of metacognition to human study habits may benefit from the behavioral distinction we make between familiarity and recollection in the rodent object recognition literature.

## Appendix

### Information gain computations: sampling across space

In this section we formalize the basic set of Bayesian computations discussed in the main text. In this case, a rat makes a decision about where to sample *x* in order to identify an observation (reward) source *h* based on observations *y*. The animal is assumed to have an observation function *p*(*y*|*h,x*) that generates expectations about the relative likelihood of a new observation *y* at a location *x* given an observation source *h*. The animal also maintains a prior for each observation source *p*(*h*|*x,I*), that incorporates past experience from *T* observations *I* = {*o*_1_,…,*o*_*T*_}. The distribution across potential observation sources is updated according to Bayes' rule:

(4) $$p(h|x,y,I)=\frac{p(y|h,x)p(h|x,I)}{\sum_{h}p(y|h,x)p(h|x,I)}$$

Now the term in the denominator *p*(*y*|*x,I*) = ∑*_h_p*(*y*|*h, x*)*p*(*h*|*x,I*) is a predictive distribution that describes how surprising a new observation is relative to previous experience. The entropy of this distribution is the sample surprise—it indicates how much diversity to expect in a new observation—and forms a baseline against which the informativeness of an observation can be assessed.

(5) $$H_{\text{surprise}}(x)=\sum_{y}p(y|x,I)\log\left(p(y|x,I)\right)$$

If we are given an observation, we can evaluate the surprise for that sample using the surprise formula without the expectation. $S_{\text{surprise}}(y|x)=p(y|x,I)\log\left(p(y|x,I)\right)$. This quantity can be used to assess how much we recognize the current sample as typical or familiar.

Now we can define the sample information in terms of how much the posterior distribution over observation (reward) sources is expected to change after obtaining another sample.

(6) $$KL_{\text{sample info}}(x)=E_{y}\left[\sum_{h}p(h|x,I)\log\left(\frac{p(h|x,y,I)}{p(h|x,I)}\right)\right]$$

Now this computation simplifies into the difference between two terms:

(7) $$KL_{\text{sample info}}(x)=\sum_{y}\sum_{h}p(y|h,x)p(h|x,I)\times \log\left(\frac{p(y|h,x)p(h|x,I)/p(y|x,I)}{p(h|x,I)}\right)$$

(8) $$=\sum_{y}\sum_{h}p(y|h,x)p(h|x,I)\times \left(\log(p(y|h,x))-\log(p(y|x,I))\right)$$

(9) $$=\;H_{\text{surprise}}(x)-H(y|h,x)$$

This computation can be interpreted as the expected information in an observation given a specific set of observations sources *H*(*y*|*h,x*) relative to the information we gain about observations when observation sources are averaged *H*_surprise_(*x*). In other words, sampling from a particular location is worthwhile only to the extent that the information derived from an observation provides more information about specific observation sources than if we simply ignored the different observation functions. This is, as a result, hierarchical rather than simple associative learning. When these computations are restricted to the animal's current position within an environment, we call it the sample information. When the computations are applied to new spatial locations, we call it the expected sample information.

### Information gain computations: sampling across reference frames

In this case, a rat makes a decision about which reference frame *h* to sample from in order to identify its location within a task *x* based on observations *y*. The animal is assumed to have an observation function *p*(*y*|*h,x*) that generates expectations about the relative likelihood of a new observation *y* at a location *x* given a reference frame *h*. The animal also maintains a spatial prior *p*(*x*|*h,I*), that incorporates past experience from *T* observations *I* = {*o*_1_,…,*o*_*T*_}. An animal can infer its location *x* given a particular map *h* using Bayes' rule:

(10) $$p(x|h,y,I)=\frac{p(h|y,x)p(y|x,I)}{\sum_{x^{\prime }}p(h|y,x^{\prime })p(y|x^{\prime },I)}$$

We now apply the same information gain computations to the inference across reference frames that we previously applied to inferences across space. The sample information is again a measure of of how much the posterior distribution changes following a sample; in this case the posterior is computed across space *x*.

(11) $$KL_{\text{sample info}}(h)\;=\;E_{y}\left[\sum_{x}p(x|y,I)\log\frac{p(x|h,y,I)}{p(x|h,I)}\right]$$

(12) $$=\;\sum_{y}\sum_{x}p(y|h,x)p(x|y,I)\log\frac{p(x|h,y,I)}{p(x|h,I)}$$

(13) $$=\;H_{\text{surprise}}(h)-H(y|h,x)$$

The term *H*_surprise_(*h*) describes how surprising an observation is given a particular map, regardless of the animal's location. This computation allows an animal to determine which reference frame is likely to provide the most information about an animal's current location.

Notably, the only differences between the expected sampling information for reference frames and the expected sampling information for space is (1) the expectation across space, rather than conditioning on space, and (2) the comparison with a different baseline, *H*_surprise_(*h*) rather than *H*_surprise_(*x*). More generally, sampling across reference frames can be construed as an attempt to acquire an observation to make an inference about location—within either a simple physical or conceptual space—while sampling across spatial locations can be construed as an attempt to acquire an observation to make an inference about observation sources—within either a simple physical or conceptual space. The consistency of these computations and their support of directed information foraging across a range of representational substrates appears to be a hallmark of hippocampal processing.

## References

[B1] Addis D. R. Wong A. T. Schacter D. L. (2007). Remembering the past and imagining the future: common and distinct neural substrates during event construction and elaboration. Neuropsychologia 45, 1363–1377 10.1016/j.neuropsychologia.2006.10.016 17126370 PMC1894691

[B2] Adhikari A. Topiwala M. A. Gordon J. A. (2010). Synchronized activity between the ventral hippocampus and the medial prefrontal cortex during anxiety. Neuron 65, 257–269 10.1016/j.neuron.2009.12.002 20152131 PMC2822726

[B3] Adhikari A. Topiwala M. A. Gordon J. A. (2011). Single units in the medial prefrontal cortex with anxiety-related firing patterns are preferentially influenced by ventral hippocampal activity. Neuron 71, 898–910 10.1016/j.neuron.2011.07.027 21903082 PMC3201792

[B4] Ainge J. A. Heron-Maxwell C. Theofilas P. Wright P. de Hoz L. Wood E. R. (2006). The role of the hippocampus in object recognition in rats: examination of the influence of task parameters and lesion size. Behav. Brain Res. 167, 183–195 10.1016/j.bbr.2005.09.005 16214239

[B5] Baillargeon R. Spelke E. S. Wasserman S. (1985). Object permanence in five-month-old infants. Cognition 20, 191–208 4064606 10.1016/0010-0277(85)90008-3

[B6] Balleine B. W. Dickinson A. (1998). Goal-directed instrumental action: contingency and incentive learning and their cortical substrates. Neuropharmacology 37, 407–419 9704982 10.1016/s0028-3908(98)00033-1

[B7] Berlyne D. E. Koenig I. D. Hirota T. (1966). Novelty, arousal, and the reinforcement of diversive exploration in the rat. J. Comp. Physiol. Psychol. 62, 222–226 5969601 10.1037/h0023681

[B8] Bethus I. Tse D. Morris R. G. M. (2010). Dopamine and memory: modulation ofthe persistence ofmemory for novel hippocampal nmda receptor-dependent paired associates. J. Neurosci. 30, 1610–1618 10.1523/JNEUROSCI.2721-09.2010 20130171 PMC6633999

[B9] Blumenthal A. Steiner A. Seeland K. Redish A. D. (2011). Effects of pharmacological manipulations of nmda-receptors on deliberation in the multiple-t task. Neurobiol. Learn. Mem. 95, 376–384 10.1016/j.nlm.2011.01.011 21296174 PMC3074592

[B10] Brown M. W. Aggleton J. P. (2001). Recognition memory: what are the roles of the perirhinal cortex and hippocampus? Nat. Rev. Neurosci. 2, 51–61 10.1038/35049064 11253359

[B11] Buckner R. L. Carroll D. C. (2007). Self-projection and the brain. Trends Cogn. Sci. 11, 49–57 10.1016/j.tics.2006.11.004 17188554

[B12] Burns B. Brock O. (2005). Toward optimal configuration space sampling, in Proceedings of Robotics: Science and Systems (Cambridge, MA: MIT Press), 1–8

[B13] Bussey T. J. Muir J. L. Aggleton J. P. (1999). Functionally dissociating aspects of event memory: the effects of combined perirhinal and postrhinal cortex lesions on object and place memory in the rat. J. Neurosci. 19, 495–502 9870977 10.1523/JNEUROSCI.19-01-00495.1999 PMC6782353

[B14] Churchwell J. C. Morris A. M. Musso N. D. Kesner R. P. (2010). Prefrontal and hippocampal contributions to encoding and retrieval of spatial memory. Neurobiol. Learn. Mem. 93, 415–421 10.1016/j.nlm.2009.12.008 20074655

[B15] Cimadevilla J. M. Wesierska M. Fenton A. A. Bures J. (2001). Inactivating one hippocampus impairs avoidance of a stable room-defined place during dissociation of arena cues from room cues by rotation of the arena. Proc. Natl. Acad. Sci. U.S.A. 98, 3531–3536 10.1073/pnas.051628398 11248112 PMC30687

[B16] Corbit L. H. Balleine B. W. (2003). Instrumental and pavlovian incentive processes have dissociable effects on components of a heterogeneous instrumental chain. J. Exp. Psychol. Anim. Behav. Process. 29, 99–106 10.1037/0097-7403.29.2.99 12735274

[B17] Cowen S. L. McNaughton B. L. (2007). Selective delay activity in the medial prefrontal cortex of the rat: contribution of sensorimotor information and contingency. J. Neurophysiol. 98, 303–316 10.1152/jn.00150.2007 17507507 PMC6257987

[B18] Day M. Langston R. Morris R. G. M. (2003). Glutamate-receptor-mediated encoding and retrieval of paired-associate learning. Nature 424, 205–209 10.1038/nature01769 12853960

[B19] de Wit S. Kosaki Y. Balleine B. W. Dickinson A. (2006). Dorsomedial prefrontal cortex resolves response conflict in rats. J. Neurosci. 26, 5224–5229 10.1523/JNEUROSCI.5175-05.2006 16687514 PMC6674252

[B20] Dix S. L. Aggleton J. P. (1999). Extending the spontaneous preference test of recognition: evidence of object-location and object-context recognition. Behav. Brain Res. 99, 191–200 10.1016/S0166-4328(98)00079-5 10512585

[B21] Dunlosky J. Hertzog C. (1998). Metacognition in Educational Theory and Practice, Chap. Training Programs to Improve Learning in Later Adulthood: Helping Older Adults Educate Themselves. Mahwah, NJ: Erlbaum

[B22] Eacott M. J. Easton A. Zinkivskay A. (2005). Recollection in an episodic-like memory task in the rat. Learn. Mem. 12, 221–223 10.1101/lm.92505 15897259

[B23] Eacott M. J. Norman G. (2004). Integrated memory for object, place, and context in rats: a possible model of episodic-like memory? J. Neurosci. 24, 1948–1953 10.1523/JNEUROSCI.2975-03.2004 14985436 PMC6730393

[B24] Easton A. Eacott M. (2008). “A new working definition of episodic memory: replacing ‘when’ with ‘which,’” in Handbook of Episodic Memory, Vol. 18, eds Dere E. Easton A. Nadel L. Huston J. P. (Amsterdam: Elsevier), 185–196

[B25] Easton A. Eacott M. J. (2010). Recollection of episodic memory within the medial temporal lobe: behavioural dissociations from other types of memory. Behav. Brain Res. 215, 310–317 10.1016/j.bbr.2009.10.019 19850082

[B26] Easton A. Zinkivskay A. Eacott M. J. (2009). Recollection is impaired, but familiarity remains intact in rats with lesions of the fornix. Hippocampus 19, 837–843 10.1002/hipo.20567 19235228

[B27] Eichenbaum H. Dudchenko P. Wood E. Shapiro M. Tanila H. (1999). The hippocampus, memory, and place cells: is it spatial memory or a memory space? Neuron 23, 209–226 10.1016/S0896-6273(00)80773-4 10399928

[B28] Eichenbaum H. Yonelinas A. P. Ranganath C. (2007). The medial temporal lobe and recognition memory. Annu. Rev. Neurosci. 30, 123–152 10.1002/hipo.20716 17417939 PMC2064941

[B29] Ennaceur A. Delacour J. (1988). A new one-trial test for neurobiological studies of memory in rats. 1, Behavioral data. Behav. Brain Res. 31, 47–59 10.1016/j.pbb.2006.08.007 3228475

[B30] Fenton A. A. Lytton W. W. Barry J. M. Lenck-Santini P. P. Zinyuk L. E. Kubik S. Bures J. Poucet B. Muller R. U. Olypher A. V. (2010). Attention-like modulation of hippocampus place cell discharge. J. Neurosci. 30, 4613–4625 10.1523/JNEUROSCI.5576-09.2010 20357112 PMC2858227

[B31] Fenton A. A. Wsierska M. Kaminsky Y. Bures J. (1998). Both here and there: simultaneous expression of autonomous spatial memories in rats. Proc. Natl. Acad. Sci. U.S.A. 95, 11493–11498 10.1073/pnas.95.19.11493 9736765 PMC21671

[B32] Fortin N. J. Agster K. L. Eichenbaum H. B. (2002). Critical role of the hippocampus in memory for sequences of events. Nat. Neurosci. 5, 458–462 10.1038/nn834 11976705 PMC4053170

[B33] Fortin N. J. Wright S. P. Eichenbaum H. B. (2004). Recollection-like memory retrieval in rats is dependent on the hippocampus. Nature 431, 188–191 10.1038/nature02853 15356631 PMC4053162

[B34] Gold J. I. Shadlen M. N. (2000). Representation of a perceptual decision in developing oculomotor commands. Nature 404, 390–394 10.1038/35006062 10746726

[B35] Gold J. I. Shadlen M. N. (2002). Banburismus and the brain: decoding the relationship between sensory stimuli, decisions, and reward. Neuron 36, 299–308 10.1016/S0896-6273(02)00971-6 12383783

[B36] Gold J. I. Shadlen M. N. (2003). The influence of behavioral context on the representation of a perceptual decision in developing oculomotor commands. J. Neurosci. 23, 632–651 12533623 10.1523/JNEUROSCI.23-02-00632.2003 PMC6741872

[B37] Granon S. Poucet B. (1995). Medial prefrontal lesions in the rat and spatial navigation: evidence for impaired planning. Behav. Neurosci. 109, 474–484 7662158 10.1037//0735-7044.109.3.474

[B38] Gupta A. (2011). Behavioral Correlates of Hippocampal Neural Sequences. Ph.D. thesis, Pittsburgh, PA: Carnegie Mellon University

[B39] Guthrie E. R. (1952). The Psychology of Learning, 2nd Edn New York, NY: Harper

[B40] Hartley T. Maguire E. A. Spiers H. J. Burgess N. (2003). The well-worn route and the path less traveled: distinct neural bases of route following and wayfinding in humans. Neuron 37, 877–888 10.1016/S0896-6273(03)00095-3 12628177

[B41] Hassabis D. Kumaran D. Maguire E. A. (2007a). Using imagination to understand the neural basis of episodic memory. J. Neurosci. 27, 14365–14374 10.1523/JNEUROSCI.4549-07.2007 18160644 PMC2571957

[B42] Hassabis D. Kumaran D. Vann S. D. Maguire E. A. (2007b). Patients with hippocampal amnesia cannot imagine new experiences. Proc. Natl. Acad. Sci. U.S.A. 104, 1726–1731 10.1073/pnas.0610561104 17229836 PMC1773058

[B43] Hasselmo M. E. (1993). Acetylcholine and learning in a cortical associative memory. Neural Comput. 5, 32–44

[B44] Hok V. Save E. Lenck-Santini P. Poucet B. (2005). Coding for spatial goals in the prelimbic/infralimbic area of the rat frontal cortex. Proc. Natl. Acad. Sci. U.S.A. 102, 4602–4607 10.1073/pnas.0407332102 15761059 PMC555486

[B45] Hu D. Amsel A. (1995). A simple test of the vicarious trial-and-error hypothesis of hippocampal function. Proc. Natl. Acad. Sci. U.S.A. 92, 5506–5509 7777539 10.1073/pnas.92.12.5506 PMC41724

[B46] Hu D. Xu X. Gonzalez-Lima F. (2006). Vicarious trial-and-error behavior and hippocampal cytochrome oxidase activity during Y-maze discrimination learning in the rat. Int. J. Neurosci. 116, 265–280 10.1080/00207450500403108 16484053

[B47] Hyman J. M. Zilli E. A. Paley A. M. Hasselmo M. E. (2005). Medial prefrontal cortex cells show dynamic modulation with the hippocampal theta rhythm dependent on behavior. Hippocampus 15, 739–749 10.1002/hipo.20106 16015622

[B48] Hyman J. M. Zilli E. A. Paley A. M. Hasselmo M. E. (2010). Working memory performance correlates with prefrontal-hippocampal theta interactions but not with prefrontal neuron firing rates. Front. Integr. Neurosci. 4:2 10.3389/neuro.07.002.2010 20431726 PMC2861479

[B49] Jackson J. Redish A. D. (2007). Network dynamics of hippocampal cell-assemblies resemble multiple spatial maps within single tasks. Hippocampus 17, 1209–1229 10.1002/hipo.20359 17764083

[B50] Johnson A. Fenton A. A. Kentros C. Redish A. D. (2009). Looking for cognition in the structure within the noise. Trends Cogn. Sci. 13, 55–64 10.1016/j.tics.2008.11.005 19135406 PMC3774297

[B51] Johnson A. Redish A. D. (2007). Neural ensembles in CA3 transiently encode paths forward of the animal at a decision point. J. Neurosci. 27, 12176–12189 10.1523/JNEUROSCI.3761-07.2007 17989284 PMC6673267

[B52] Jones M. Wilson M. (2005). Theta rhythms coordinate hippocampal-prefrontal interactions in a spatial memory task. PLoS Biol. 3:e402 10.1371/journal.pbio.0030402 16279838 PMC1283536

[B53] Kaesermann H. P. (1986). Stretched attend posture, a non-social form of ambivalence, is sensitive to a conflict-reducing drug action. Psychopharmacology (Berl.) 89, 31–37 2874583 10.1007/BF00175185

[B54] Kelemen E. Fenton A. A. (2010). Dynamic grouping of hippocampal neural activity during cognitive control of two spatial frames. PLoS Biol. 8:e1000403 10.1371/journal.pbio.1000403 20585373 PMC2889929

[B55] Kumaran D. Maguire E. A. (2006). An unexpected sequence of events: mismatch detection in the human hippocampus. PLoS Biol. 4:e424 10.1371/journal.pbio.0040424 17132050 PMC1661685

[B56] Kyd R. J. Bilkey D. K. (2003). Prefrontal cortex lesions modify the spatial properties of hippocampal place cells. Cereb. Cortex 13, 444–451 10.1093/cercor/13.5.444 12679291

[B57] Li L. Miller E. K. Desimone R. (1993). The representation of stimulus familiarity in anterior inferior temporal cortex. J. Neurophysiol. 69, 1918–1929 8350131 10.1152/jn.1993.69.6.1918

[B58] Loewenstein G. (1994). The psychology of curiosity: a review and reinterpretation. Psychol. Bull. 116, 75–98

[B59] Maviel T. Durkin T. P. Menzaghi F. Bontempi B. (2004). Sites of neocortical reorganization critical for remote spatial memory. Science 305, 96–99 10.1126/science.1098180 15232109

[B60] Mazurek M. E. Roitman J. D. Ditterich J. Shadlen M. N. (2003). A role for neural integrators in perceptual decision making. Cereb. Cortex 13, 1257–1269 10.1093/cercor/bhg097 14576217

[B61] Metcalfe J. (2009). Metacognitive judgments and control of study. Curr. Dir. Psychol. Sci. 18, 159–163 10.1111/j.1467-8721.2009.01628.x 19750138 PMC2742428

[B62] Metcalfe J. Kornell N. (2005). A region of proximal learning model of study time allocation. J. Mem. Lang. 52, 463–477

[B63] Morris R. G. M. (2006). Elements of a neurobiological theory of hippocampal function: the role of synaptic plasticity, synaptic tagging and schemas. Eur. J. Neurosci. 23, 2829–2846 10.1111/j.1460-9568.2006.04888.x 16819972

[B64] Morris R. G. M. Garrud P. Rawlins J. N. P. O'Keefe J. (1982). Place navigation impaired in rats with hip pocampal lesions. Nature 297, 681–683 7088155 10.1038/297681a0

[B65] Muenzinger K. F. (1938). Vicarious trial and error at a point of choice: a general survey of its relation to learning efficiency. J. Genet. Psychol. 53, 75–86

[B66] Mumby D. G. Gaskin S. Glenn M. J. Schramek T. E. Lehmann H. (2002). Hippocampal damage and exploratory preferences in rats: memory for objects, places, and contexts. Learn. Mem. 9, 49–57 10.1101/lm.41302 11992015 PMC155935

[B67] Nadel L. Moscovitch M. (1997). Memory consolidation, retrograde amnesia and the hippocampal complex. Curr. Opin. Neurobiol. 7, 217–227 10.1002/hipo.20607 9142752

[B69] O'Keefe J. Nadel L. (1978). The Hippocampus as a Cognitive Map. Oxford: Clarendon Press

[B70] Ostlund S. B. Balleine B. W. (2005). Lesions of medial prefrontal cortex disrupt the acquisition but not the expression of goal-directed learning. J. Neurosci. 25, 7763–7770 10.1523/JNEUROSCI.1921-05.2005 16120777 PMC6725247

[B71] Packard M. G. McGaugh J. L. (1996). Inactivation of hippocampus or caudate nucleus with lidocaine differentially affects expression of place and response learning. Neurobiol. Learn. Mem. 65, 65–72 10.1006/nlme.1996.0007 8673408

[B72] Redish A. D. (1999). Beyond the Cognitive Map: From Place Cells to Episodic Memory. Cambridge, MA: MIT Press

[B73] Rich E. L. Shapiro M. (2009). Rat prefrontal cortical neurons selectively code strategy switches. J. Neurosci. 29, 7208–7219 10.1162/jocn.2009.21007 19494143 PMC3229282

[B74] Rich E. L. Shapiro M. L. (2007). Prelimbic/infralimbic inactivation impairs memory for multiple task switches, but not flexible selection of familiar tasks. J. Neurosci. 27, 4747–4755 10.1523/JNEUROSCI.0369-07.2007 17460087 PMC6672999

[B75] Rolls E. T. Cahusac P. M. Feigenbaum J. D. Miyashita Y. (1993). Responses of single neurons in the hippocampus of the macaque related to recognition memory. Exp. Brain Res. 93, 299–306 8491268 10.1007/BF00228398

[B76] Santos L. R. (2004). Core knowledges: a dissociation between spatiotemporal knowledge and contact-mechanics in a non-human primate? Dev. Sci. 7, 167–174 10.1111/j.1467-7687.2004.00335.x 15320376

[B77] Schacter D. L. Addis D. R. (2007). The cognitive neuroscience of constructive memory: remembering the past and imagining the future. Philos. Trans. R. Soc. Lond. B Biol. Sci. 362, 773–786 10.1098/rstb.2007.2087 17395575 PMC2429996

[B78] Schacter D. L. Addis D. R. Buckner R. L. (2007). Remembering the past to imagine the future: the prospective brain. Nat. Rev. Neurosci. 8, 657–661 10.1038/nrn2213 17700624

[B79] Shadlen M. N. Newsome W. T. (2001). Neural basis of a perceptual decision in the parietal cortex (area lip) of the rhesus monkey. J. Neurophysiol. 86, 1916–1936 11600651 10.1152/jn.2001.86.4.1916

[B80] Siapas A. G. Lubenov E. V. Wilson M. A. (2005). Prefrontal phase locking to hippocampal theta oscillations. Neuron 46, 141–151 10.1016/j.neuron.2005.02.028 15820700

[B81] Spelke E. S. Kinzler K. D. (2007). Core knowledge. Dev. Sci. 10, 89–96 10.1111/j.1467-7687.2007.00569.x 17181705

[B82] Squire L. R. Alvarez P. (1995). Retrograde amnesia and memory consolidation: a neurobiological perspective. Curr. Opin. Neurobiol. 5, 169–177 10.1016/0959-4388(95)80023-9 7620304

[B83] Squire L. R. Stark C. E. L. Clark R. E. (2004). The medial temporal lobe. Annu. Rev. Neurosci. 27, 279–306 10.1016/j.bbr.2007.12.018 15217334

[B84] Summerfield J. J. Hassabis D. Maguire E. A. (2010). Differential engagement of brain regions within a ‘core’ network during scene construction. Neuropsychologia 48, 1501–1509 10.1016/j.neuropsychologia.2010.01.022 20132831 PMC2850391

[B85] Teng E. Squire L. R. (1999). Memory for places learned long ago is intact after hippocampal damage. Nature 400, 675–677 10.1038/23276 10458163

[B86] Tolman E. C. (1939). Prediction of vicarious trial and error by means of the schematic sowbug. Psychol. Rev. 46, 318–336

[B87] Tolman E. C. (1948). Cognitive maps in rats and men. Psychol. Rev. 55, 189–208 18870876 10.1037/h0061626

[B88] Tolman E. C. (1954). Freedom and the cognitive mind. Am. Psychol. 9, 536–538

[B89] Tse D. Langston R. F. Kakeyama M. Bethus I. Spooner P. A. Wood E. R. Witter M. P. Morris R. G. M. (2007). Schemas and memory consolidation. Science 316, 76–82 10.1016/j.nlm.2007.09.007 17412951

[B90] Tse D. Takeuchi T. Kakeyama M. Kajii Y. Okuno H. Tohyama C. Bito H. Morris R. G. M. (2011). Schema-dependent gene activation and memory encoding in neocortex. Science 333, 891–895 10.1126/science.1205274 21737703

[B91] Voss J. L. Galvan A. Gonsalves B. D. (2011a). Cortical regions recruited for complex active-learning strategies and action planning exhibit rapid reactivation during memory retrieval. Neuropsychologia 49, 3956–3966 10.1016/j.neuropsychologia.2011.10.012 22023912 PMC3223278

[B92] Voss J. L. Gonsalves B. D. Federmeier K. D. Tranel D. Cohen N. J. (2011b). Hippocampal brain-network coordination during volitional exploratory behavior enhances learning. Nat. Neurosci. 14, 115–120 10.1038/nn.2693 21102449 PMC3057495

[B93] Voss J. L. Warren D. E. Gonsalves B. D. Federmeier K. D. Tranel D. Cohen N. J. (2011c). Spontaneous revis-itation during visual exploration as a link among strategic behavior, learning, and the hippocampus. Proc. Natl. Acad. Sci. U.S.A. 108, E402–E409 10.1073/pnas.1100225108 21768385 PMC3150890

[B94] Warburton E. C. Aggleton J. P. (1999). Differential deficits in the morris water maze following cytotoxic lesions ofthe anterior thalamus and fornix transection. Behav. Brain Res. 98, 27–38 10.1016/S0166-4328(98)00047-3 10210519

[B95] Wesierska M. Dockery C. Fenton A. A. (2005). Beyond memory, navigation, and inhibition: behavioral evidence for hippocampus-dependent cognitive coordination in the rat. J. Neurosci. 25, 2413–2419 10.1523/JNEUROSCI.3962-04.2005 15745968 PMC6726107

[B96] Winters B. Bussey T. (2005). Transient inactivation of perirhinal cortex disrupts encoding, retrieval, and consolidation of object recognition. J. Neurosci. 25, 52–61 10.1523/JNEUROSCI.3827-04.2005 15634766 PMC6725205

[B97] Winters B. Forwood S. Cowell R. Saksida L. Bussey T. (2004). Double dissociation between the effects of peri-postrhinal cortex and hippocampal lesions on tests of object recognition and spatial memory: heterogeneity of function within the temporal lobe. J. Neurosci. 24, 5901–5908 10.1523/JNEUROSCI.1346-04.2004 15229237 PMC6729235

[B98] Xiang J. Z. Brown M. W. (1998). Differential neuronal encoding of novelty, familiarity and recency in regions of the anterior temporal lobe. Neuropharmacology 37, 657–676 9705004 10.1016/s0028-3908(98)00030-6

[B99] Yang T. Shadlen M. N. (2007). Probabilistic reasoning by neurons. Nature 447, 1075–1080 10.1038/nature05852 17546027

[B100] Yonelinas A. P. (2001). Components of episodic memory: the contribution of recollection and familiarity. Philos. Trans. R. Soc. Lond. B Biol. Sci. 356, 1363–1374 10.1098/rstb.2001.0939 11571028 PMC1088520

[B101] Zhou W. Crystal J. D. (2009). Evidence for remembering when events occurred in a rodent model of episodic memory. Proc. Natl. Acad. Sci. U.S.A. 106, 9525–9529 10.1073/pnas.0904360106 19458264 PMC2695044

[B102] Zhu X. O. Brown M. W. Aggleton J. P. (1995). Neuronal signalling of information important to visual recognition memory in rat rhinal and neighbouring cortices. Eur. J. Neurosci. 7, 753–765 7620624 10.1111/j.1460-9568.1995.tb00679.x

[B103] Zola-Morgan S. Squire L. R. (1990). The primate hippocampal formation: evidence for a time-limited role in memory storage. Science 250, 288–290 10.1126/science.2218534 2218534

